# Using Artificial Intelligence for High-Volume Identification of Silicosis and Tuberculosis: A Bio-Ethics Approach

**DOI:** 10.5334/aogh.3206

**Published:** 2021-07-01

**Authors:** Jerry M. Spiegel, Rodney Ehrlich, Annalee Yassi, Francisco Riera, James Wilkinson, Karen Lockhart, Stephen Barker, Barry Kistnasamy

**Affiliations:** 1 School of Population and Public Health, The University of British Columbia, Vancouver, BC, Canada; 2 School of Public Health and Family Medicine, University of Cape Town, Cape Town, South Africa; 3 IMC Worldwide Inc., London, UK; 4 Brink, London, UK; 5 National Department of Health, Johannesburg, South Africa

## Abstract

Although Artificial Intelligence (AI) is being increasingly applied, considerable distrust about introducing “disruptive” technologies persists. Intrinsic and contextual factors influencing where and how such innovations are introduced therefore require careful scrutiny to ensure that health equity is promoted. To illustrate one such critical approach, we describe and appraise an AI application – the development of computer assisted diagnosis (CAD) to support more efficient adjudication of compensation claims from former gold miners with occupational lung disease in Southern Africa. In doing so, we apply a bio-ethical lens that considers the principles of beneficence, non-maleficence, autonomy and justice and add explicability as a core principle. We draw on the AI literature, our research on CAD validation and process efficiency, as well as apprehensions of users and stakeholders. Issues of concern included AI accuracy, biased training of AI systems, data privacy, impact on human skill development, transparency and accountability in AI use, as well as intellectual property ownership. We discuss ways in which each of these potential obstacles to successful use of CAD could be mitigated. We conclude that efforts to overcoming technical challenges in applying AI must be accompanied from the onset by attention to ensuring its ethical use.

## Introduction

At a moment when the need to turn to online solutions is greater than ever, and artificial Intelligence (AI) is widely heralded as transformative, considerable distrust about introducing such technology persists [1]. The COVID-19 pandemic has sharpened attention to the potential for innovative uses of AI to provide timely access to information while minimizing personal exposures to the virus [2]. To illustrate what this can mean for a marginalized population, we focus here on an AI application in the form of computer aided detection (CAD) of silicosis and tuberculosis in active and former gold miners suffering from occupational lung disease in Southern Africa.

Since AI was first introduced in the 1940s, its uses have grown to become commonplace - from speech recognition to modern banking as well as myriad health-related purposes [3]. The driver of specific applications has primarily been the promise of improving the proficiency and efficiency of operations that draw on labour-intensive and imperfect human judgement for making decisions. But, as is common when new “disruptive” technologies are initiated, resistance may be provoked among those affected [4]. With this in mind, the use of machine learning to address global health equity challenges merits particular consideration not only of its benefits in identifying existing yet sometimes unobserved patterns to generate knowledge out of complex underlying data [5], but also of possible undesirable consequences of such application.

Within clinical medicine, AI has been widely used with success, such as for the early diagnosis and treatment of stroke [6], breast cancer detection [7], assessment of skin lesions [8] as well as analysis of chest x-rays (CXRs) for lung cancer [9]. As experience has been gained in implementing AI solutions, approaches for ensuring its responsible development have been advanced [10]. In this regard, a global health lens on harnessing AI technology to improve the welfare of historically marginalized populations should consider not only the risks of misuse or overuse, but also the benefits of mitigation measures that might be applied to avert the “opportunity cost” of underuse [11].

There has been active exploration of the potential benefits of harnessing such new technology in public health, including for the attainment of in the Sustainable Development Goals [12] and challenges such as tuberculosis (TB), a disease of huge global health concern [13]. In respect of TB, AI advancements have indeed been made through the use of artificial neural networks in computer-aided radiological detection (CAD) [14]. Algorithms are now available to accomplish tasks previously undertaken laboriously by human review of each image [15], with studies beginning to show equivalence when compared to human expert readers [16,17]. However, before we began this project, none of the existing CAD TB models developed to date had been validated for use in populations with high rates of both TB and silicosis.

## Context

In Southern Africa, a powerful legacy of social injustice has been the prevalence of occupational lung disease, particularly silicosis and TB, among the miners who produced so much wealth for the global economy [18,19,20,21,22]. A dominant feature of the labour system for South Africa’s gold mines has been oscillating migrant labour, with large numbers of migrant miners having left families in Botswana, Lesotho, Mozambique, Eswatini and elsewhere in Southern Africa, along with miners from regions within South Africa such as the Eastern Cape, to work in mines far from home [21,22].

Besides causing silicosis [18,19], silica inhalation in underground mining and the resultant silicosis increases the risk of active pulmonary TB [23,24] as well as the risk of post-TB fibrosis [24,25]. TB is further amplified in this population by the migrant labour system, high prevalence of HIV infection, and crowded transport and living conditions [18,20,21,22,26]. Importantly, TB can also result in lung changes that mask the appearance of silicosis or mimic it on the CXR, creating problems for diagnosis [25].

Although democracy was established in South Africa in 1994, failure to provide health surveillance for ex-miners in the post-apartheid era has left potentially hundreds of thousands throughout Southern Africa at risk of these diseases with limited or no access to health assessment and treatment, nor to related financial compensation [19,27,28]. To address previously unmet needs, South Africa’s statutory compensation agencies, in the form of the Medical Bureau for Occupational Diseases (MBOD) and the Compensation Commissioner for Occupational Diseases (CCOD), have introduced mobile clinics and One Stop Centres to improve access to compensation for occupational lung disease [29]. In the civil arena, lawyers acting on behalf of gold miners, active and former, have recently pursued a successful class action suit for silicosis and tuberculosis against six gold mining companies [30].

Despite these efforts to improve access to medical examination and the submission of claims, a severe bottleneck has persisted in the medical adjudication processing of submitted claims within the statutory compensation system [29]. The average monthly backlog in claims in 2019 was over 12,000, while the average delay between the primary medical examination and certification for compensation from April 2019 to October 2020 ran at 577 days (Andre Fourie, personal communication). Besides the high volume of submissions, an important cause of the long delay is that relevant legislation requires a radiologist and a four-person medical panel to certify compensability or otherwise in all claims. Fewer than 28% of claims turn out to be eligible for compensation (ibid.). Also, 45% are claims for wage replacement for active TB in working miners (“temporary disability”), for which a much lower intensity of medical assessment is arguably needed than for permanent impairment or disease. However, all claims currently have to receive the same degree of assessment (Dr. N. Mtshali, MBOD Director, personal communication).

The project in which the authors are engaged was based on the expectation that triage of claims not requiring this resource-intensive five-person assessment procedure into a less resource-intensive but still accurate and fair process could greatly improve the efficiency of statutory processing of claims of ex-miners. One of the options explored for such triage was pre-classification of CXRs using CAD. The work therefore involved a validation study using carefully selected CXRs of four commercial CAD systems pre-trained on TB and silicosis against the radiologist and four-person panel [31]. This research indicated that CAD would be best used in this context to identify CXRs without silicosis or TB by offering a high degree of sensitivity (few false negatives) [32]. This study was followed up with a second validation trial using unselected CXRs from field screening of ex-miners against expert readers outside the MBOD system [33]. This “real world” study yielded lower specificities for a given sensitivity, entailing a greater cost of false positives than suggested by the earlier trial [33]. Interpretation of these results with a view to application is underway with the active involvement of stakeholders.

**Figure 1:**
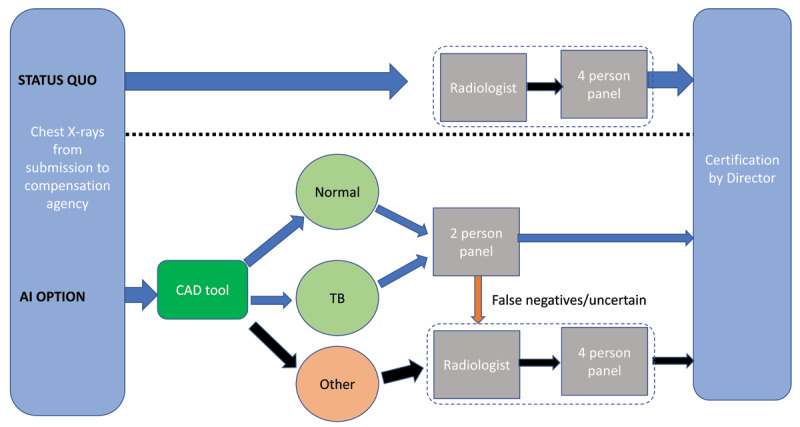
Alternative approaches for assessing compensation claim chest x-rays.

In parallel, the MBOD instituted a two-tier system – one with the radiologist and four-person panel as before, and the other made up of two *two-person panels* to assess claims requiring a lower level of assessment; specifically, those not likely to have a compensable disease and those with TB, likely to be wage loss claims rather than needing assessment for permanent impairment. The further role of the two-person panels was to escalate potentially compensable claims for silicosis or TB permanent disease or impairment to the higher panel. The project (summarized visually in ***Figure 1***) has so far shown promise in relieving pressure on the four-person panel and reducing the backlog in claims processing (Dr. Nhlanhla Mtshali, personal communication). The role and sustainability of CAD in practice, however, is the subject of ongoing research.

## Viewpoint objective

In the process of undertaking this project, a number of broader systemic questions emerged in collaboration with our partners, issues which included but went beyond the accuracy of CAD. We identified these as being mainly of an ethical nature. Given the marked interest and growth in applying CAD in radiology, we believe that attention needs to be paid to ensuring that this technology is used in a way that is ethically sound and clearly introduced as such to those involved [34,35]. We have therefore synthesized our observations with reference to the recognized biomedical ethics pillars [36] of beneficence (promotion of wellbeing); non-maleficence (avoidance of harm); autonomy (respecting the power to decide); and justice (promotion of solidarity)as well as an appreciation of global health as a frame for promoting health equity worldwide [37]. We have added a fifth consideration, *explicability* (understanding and holding to account the AI decision-making processes) proposed by Floridi et al. [11] to draw attention to the importance of involvement of stakeholders when implementation is being planned [11].

This viewpoint highlights questions that we believe decision-makers and stakeholders should ask when undertaking development of CAD [38], and AI applications more broadly. Our perspective is based on our CAD research; the general AI implementation literature; clinical site visits; and a workshop with those responsible for the statutory compensation process and members of the Tshiamiso Trust, set up following the class action suit [30]. It also draws on our interaction with involved health practitioners, government decision-makers, AI vendors, and interested parties representing both workers and mining company interests, including in a final Zoom-workshop of over 50 participants to discuss the results presented here [33,38].

## Applying bio-ethical principles to the introduction of an AI system

**Figure 2:**
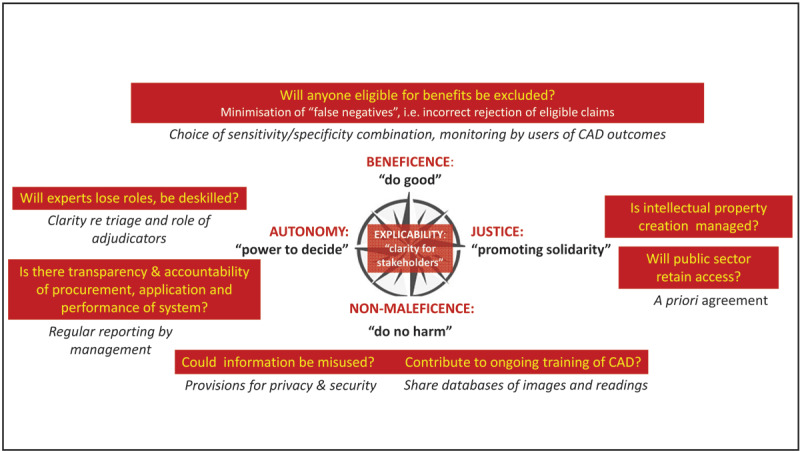
Reflecting on principles of bio-ethics in relation to our CAD application.

***Figure 2*** provides a schematic of the five bio-ethical principles considered, with examples of questions to be asked in the introduction of a CAD system, and responsive actions to promote ethical outcomes. ***Table 1*** provides our summary of the main arguments raised against using AI together with a consideration of counter-arguments and further implications. We discuss these below.

**Table 1:** The case for and against use of an AI application (Computer Assisted Diagnosis of silicosis and tuberculosis) for assessment of claims for occupational lung disease in miners.


THE CASE AGAINST USING AI *(CONCERNS ENCOUNTERED IN THIS CASE)*	COUNTER-ARGUMENTS OR MITIGATION	ACTIONS NEEDED

**BENEFICENCE: do good**		

AI is of value if accurate. *(Concern about rejection of legitimate claims)*	Rigorous monitoring and evaluation would need to continue after introduction to ensure satisfactory sensitivity and specificity – so that CAD systems continue to improve with feedback.	Committing resources for ongoing monitoring and evaluation real world applications.

**NON-MALEFICENCE: do no harm**		

Privacy and security of personal information could be compromised. *(Concern that privacy will be breached in handling)*	Privacy and security of data are equally of concern in systems that do not use AI. Arguably data protection measures could more easily be put in place in data-driven systems.	Protocols covering access to data need to be written/agreed upon by all users.

AI training could be subject to bias – for example, if trained against “gold standards” that are themselves inaccurate.*(Concern about bias in development of AI)*	The AI systems need to be repeatedly assessed for accuracy against different and independent “gold standards” to avoid the biases of any one group of experts.	Willingness to share databases alongside ongoing resource commitment.

**AUTONOMY: power to decide**		

Reliance on AI could decrease availability of needed skilled experts and lead to de-skilling of clinical judgment.*(Concern that skilled experts will be displaced)*	If the system is used for triage, rather than replacement of human expertise, it would serve to make specialists’ time more efficient and reduce the cost burden of specialist services. Specialists would need to understand the limits of AI to avoid over-reliance on the AI.	In the compensation context, there needs to be a strong understanding amongst all stakeholders that the intent is for triage rather than screening out. Ongoing monitoring is needed to ensure that complacency doesn’t take hold. Also, specialists should be trained to expect and look for false negatives and false positives.

Transparency could be diminished such that users are disempowered.*(Concern that there will be less accountability)*	Assumptions inherent in the systems should be transparent, including accuracy, i.e. sensitivity and specific for each type of assessment. Accountability would need to remain with clinicians who use the system and the medical professionals who sign off on cases.	Sustained commitment to openness and transparency is needed.

**JUSTICE: promote solidarity**		

Proprietary ownership of AI could make it prohibitively expensive for the public sector.*(Concern that high cost of AI could limit its use)*	As public sector data are being used to train AI systems, *a priori* agreement would need to be signed off to ensure that the cost to the public sector is reasonable.	A change of payment provisions may be needed, as AI companies depend on royalty revenue unless access provisions are specified for public interest uses.


### Beneficence: Delivery of benefit

Uncertainty about the CAD system’s ability to accurately classify TB and silicosis to support the timely decision-making required within the compensation process is a primary source of mistrust. Particular apprehension among the advocates for ex-miner interests is the prospect of CAD generating “false negative” disease classification readings that would result in unjust rejection of claims.

As CAD use for TB screening and triage has increased and different proprietary systems enter the market, concerns about accuracy have come to the fore [17,39]. A recent systematic review of studies of CAD diagnostic accuracy identified a number of technical elements that should be included in studies to improve clinical applicability of future CAD studies [17]. These include describing how the CXRs were selected for training and testing; using CXRs from distinct databases for training and testing; employing a microbiologic reference standard in order to evaluate the accuracy of CAD in the case of TB; and reporting the threshold score to differentiate between a positive or negative CXR alongside a description of how this was determined.

In this project, the best performing CAD system in the first trial was found to have a sensitivity and specificity each of 98.2% when identifying the presence of “tuberculosis or silicosis,” offering high promise of an effective screening tool [31]. In the repeated trial with a set of CXRs drawn from a more realistic field setting, and a different set of readers, the sensitivity and specificity of the same AI system fell to 90.1% and 80.3%, which are still promising, however. These findings underscore the importance of using appropriate training and validation sets of CXRs, and the need for continual monitoring of CAD performance in use and in iterative machine learning. This project is the first to include CAD for silicosis, which would find application in the many silica exposed populations globally.

Differences in silicosis and TB reading between readers found in the second trial were consistent with what has been found in other inter-reader studies [40,41]. This reminds us that human diagnosis is also prone to error, and that in this regard the use of AI for silicosis could potentially provide more consistent adjudication than that resulting from decisions left to diverse practitioners with variable skill levels.

With regard to the threshold for a positive disease classification, there is a desirable “bias” in the compensation context. This is to set the system to minimize false negatives (although at the cost of more false positives). This needs to be accompanied by awareness on the part of the medical adjudicators of the margin of machine error they are working with and the corresponding need for monitoring and corrective learning [32].

### Non-Maleficence: Avoidance of harm

As the machine learning basis of AI applications hinges on commercial entities and researchers gaining access to large sets of health data from thousands of individuals, there is need to maintain the privacy and security of such data, even if eventually used anonymously for training. Recent data breaches of large swathes of personal health data have shown that researchers and those working with data using AI need to be cognizant of the risks [34]. In focusing on the ethical and legal issues related to AI in radiology, the Canadian Association of Radiologists Artificial Intelligence Working Group [35], emphasizes that:

*Tempering the incentive to share patient data for AI training is the potential threat of patient privacy and confidentiality breaches. The collection, storage, and use of bulk medical data present additional challenges related to social acceptability and public perception, legislative obstacles, information technology barriers, and the risk of breaches in data security* [42].

This domain has recently been regulated by the South African state in the form of the Privacy of Private Information Act [43]. Within the compensation system, this legislation will put additional pressure on those responsible for managing large archives of CXRs – in the arrangements with CAD providers as well as medical practitioners accessing these databases.

More generally with regard to AI application, the problem of more insidious and unrecognized biases has been raised. Dwivedi and colleagues caution that:

*As human developers have written the algorithms that are used within AI based systems, it should not be a surprise that a number of inherent biases have slipped through into decision making systems. The implication for bias within AI systems is significant as people may end up being disenfranchised by incorrect logic and decision making* [44].

As a specific example of this, we note that fewer than 10% of the high-risk workforce are women, and less than 2% of the claims in the MBOD database for silicosis and/or TB to date are from women (as women were recruited into production jobs relatively recently). In light of this, samples for machine learning need to start including CXR images from women to ascertain if the CAD performance on female chest images is significantly different from its accuracy on male images.

### Autonomy: Maintaining the power to make choices

Striking the right balance between human agency and the value that can be gained from AI requires consideration of how specific options might be adopted as well as the potential for undermining the human capacities needed for oversight of AI use.

One of the apprehensions about AI is that it could lead to de-skilling, as relevant professionals come to rely more heavily on the AI than on their own clinical judgement thereby undermining capacities for independent assessment. As defined by Topol, “automation bias” occurs when humans tend to accept machine decisions even when they are wrong [45]. However, it has also been shown that clinicians can be trained to avoid this [46]. Medical professionals need to understand the limits of AI and remain vigilant. In the compensation context, they should be prompted to expect and look for false negatives and false positives, and improve their skills in the process of checking the CAD [7].

It follows that it is necessary to have clearly established protocols as to next steps when the medical adjudicator disagrees with the CAD assessment. In other contexts, keeping up the diagnostic training of radiologists, occupational medical specialists and others who may be involved in the detection of occupational lung disease and TB, is needed. In resource poor settings where there is no radiologist back-up, the use of CAD by primary level practitioners responsible for screening for TB and silicosis has the potential to upskill them as well.

For autonomy, transparency in data acquisition, and indeed throughout the entire data pipeline [47], is needed to ensure full disclosure and representation. A recent study in this regard observed that data users such as government policy stakeholders as well as those speaking on behalf of hospital managers and/or doctors see the lack of transparency of AI algorithms as one of the biggest impediments to uptake of AI [48]. In contrast, this was not an expressed concern of data processors.

### Justice: Promoting benefit and solidarity

Even when the ability of the AI technology to improve health equity has been demonstrated, the process by which an AI application is introduced and implemented still merits attention, recognizing that the need for investment in such pursuits may well prompt a comparative neglect of economically marginalized parties’ priorities. For example, while it is not uncommon for AI technology itself to be developed with the aid of public sector resources, either directly in the development process or through sharing of datasets for training purposes or validation, subsequent commercialization and implementation by private companies can lead to barriers to subsequent access by potential beneficiaries with limited economic resources. Although open source software has been used in the development of CAD systems, commercial systems predominate in clinical applications [17], and are the basis of the experience described in this piece.

This problem is analogous to that of a new drug being unaffordable to those who participated in its research trials. The business model of private enterprises requires them to maximize the return on their investment, creating for the market leaders a potential for monopoly dominance in supply and pricing. To promote social good as the focus and rationale for innovation, ways need to be sought to keep choices open and costs affordable for the public sector. Failure to consider this could undermine trust in potentially beneficial applications.

There is also a need to consider the situation where innovative technology fails to be used. In situations where market demand is too weak to drive investment in AI application and where the responsible public institutions have not responded, the failure to use available technology could be seen as maintaining inequalities. In South Africa, there has been considerable social pressure for redistributive justice for gold-miners – via political channels and via civil litigation [49]. However, this solidarity needs to be carried though to ensure that eligible claimants, many of whom living in poverty, actually see the funds put into their bank accounts. This puts the spotlight on the timely use of technological innovations to benefit those in need.

## Discussion

Introducing new technology in healthcare is thus far more than a technical efficacy matter, as a much wider set of issues must be considered regarding how implementation can alter decision-making and related social processes. There may be sharp differences in how different stakeholders perceive the changed distribution of benefits and other consequences when established patterns are disrupted [4]. Moreover, in lower-income settings marked by power inequalities, negative effects may be pronounced [50]. Recognizing these concerns, alongside the benefits, is needed to optimally achieve the potential of AI solutions.

In 2018, the Canadian Institutes of Health Research (CIHR) held workshops related to the theme of AI and health equity [47]. In addition to considering broad solution areas where new application could be pursued, attention was drawn to the degree to which innovations, as they are developed and implemented, respond to the needs of outliers and individuals from under-represented groups rather than the usual voices:

*AI & ML (machine learning) researchers should consider outliers and individuals from underrepresented populations by adopting a ‘lawn mower of justice’ (see Dr. Jutta Treviranus’s work) [51] approach which attempts to increase the influence of outlier and non-common voices by limiting the weight we put on data from the most represented groups (i.e. set a maximum number of data points that can be analyzed from any one group). …. Researchers should engage ethicists, people with lived experiences, and communities affected by research in developing AI tools from the beginning/design stages*.

In our case, work is still underway to ensure that the algorithms developed are applicable to the specific disease features of this population of gold miners. As recently noted by Racine and colleagues [52], applications of AI in imaging and diagnoses, risk analysis and health information management, amongst others [45,52], are being widely adopted, with predicted benefits including the promise of decreased healthcare costs and inefficiency. The urgency needed to address legacies of health and social injustice may thus provide a powerful incentive for adopting AI-based technology. However, there is a need to accompany empirical evidence of the validity/reliability of this technology with open discussion of ethical implications of potential biases as well as full transparency about data inclusion/exclusion [53,54], the decision-making process, and the ownership of the data upon which algorithms are developed [45,52].

Floridi et al. emphasize that in considering the case for applying AI, *explicability* should be added as a fifth principle to complement the traditional four principles of beneficence, non-maleficence, autonomy and justice that we have discussed above [11]. This requires providing clear communication on the questions, “how does it work?” and “who is responsible for the way it works?” The process of ensuring that solutions are developed with clarity and direct consideration of the concerns of stakeholders is fundamental to the integration of a disruptive technology in the area of health assessment where social injustice issues are central to its application.

## Conclusion

It is timely to take stock of barriers that might undermine the advancement of AI innovation on behalf of those who have been socially marginalized. On the eve of the French Revolution, democratic rights champion Jean-Jacques Rousseau provocatively argued *against* pursuing “science and the arts” in society if doing so would be corrupted by narrow interests contrary to the public good [55]. Similar lines of argumentation have even been invoked to question the terms for promoting public health in general [56].

As discussed in this piece, there are mitigating measures that could be put in place to address each of the concerns raised about a particular AI application, which, we argue, are necessary to consider given the need to provide compensation to ex-miners in Southern Africa who have developed lung disease while creating enormous wealth. Within a global perspective, analogous opportunities for AI to provide benefit for marginalized populations should be similarly considered. While investment is needed to solve technical deficiencies, effort is also needed to ensure ethical application so that social justice is served.

## References

[1] Helbing D, Frey BS, Gigerenzer G, et al. Will democracy survive big data and artificial intelligence? Towards digital enlightenment. Springer. 2019; 73–98. DOI: 10.1007/978-3-319-90869-4_7

[2] Naudé W. Artificial intelligence vs COVID-19: limitations, constraints and pitfalls. AI & Society. 2020; 35: 761–765. DOI: 10.1007/s00146-020-00978-0 PMC7186767 32346223

[3] Buchanan BG. A (very) brief history of artificial intelligence. AI Magazine. 2005; 26(4): 53–53.

[4] Danneels E. Disruptive technology reconsidered: A critique and research agenda. Journal of Product Innovation Management. 2004; 21(4): 246–258. DOI: 10.1111/j.0737-6782.2004.00076.x

[5] Murdoch WJ, Singh C, Kumbier K, Abbasi-Asl R, Yu B. Interpretable machine learning: Definitions, methods, and applications. arXiv preprint arXiv:190104592. 2019. 10.1073/pnas.1900654116 PMC6825274 31619572

[6] Murray NM, Unberath M, Hager GD, Hui FK. Artificial intelligence to diagnose ischemic stroke and identify large vessel occlusions: A systematic review. Journal of Neurointerventional Surgery. 2020; 12(2): 156–164. DOI: 10.1136/neurintsurg-2019-015135 31594798

[7] Carter SM, Rogers W, Win KT, Frazer H, Richards B, Houssami N. The ethical, legal and social implications of using artificial intelligence systems in breast cancer care. The Breast. 2020; 49: 25–32. DOI: 10.1016/j.breast.2019.10.001 31677530 PMC7375671

[8] Marka A, Carter JB, Toto E, Hassanpour S. Automated detection of nonmelanoma skin cancer using digital images: A systematic review. BMC Medical Imaging. 2019; 19(1): 21. DOI: 10.1186/s12880-019-0307-7 30819133 PMC6394090

[9] Dubey AK, Gupta U, Jain S. Epidemiology of lung cancer and approaches for its prediction: A systematic review and analysis. Chinese Journal of Cancer. 2016; 35(1): 71. DOI: 10.1186/s40880-016-0135-x 27473753 PMC4967338

[10] Université de Montréal. Montreal Declaration for Responsible AI. https://www.montrealdeclaration-responsibleai.com/the-declaration. Accessed: 2 March 2020; 2017.

[11] Floridi L, Cowls J, Beltrametti M, et al. AI4People—An ethical framework for a good AI society: Opportunities, risks, principles, and recommendations. Minds and Machines. 2018; 28(4): 689–707. DOI: 10.1007/s11023-018-9482-5 30930541 PMC6404626

[12] Chui M, Harrysson M, Manyika J, et al. Applying Artificial Intelligence for Social Good. https://www.mckinsey.com/featured-insights/artificial-intelligence/applying-artificial-intelligence-for-social-good#: McKinsey & Company; 12 2018.

[13] Zumla A, Petersen E. The historic and unprecedented United Nations General Assembly High Level Meeting on Tuberculosis (UNGA-HLM-TB)—‘United to End TB: An Urgent Global Response to a Global Epidemic’. International Journal of Infectious Diseases. 2018; 75: 118–120. DOI: 10.1016/j.ijid.2018.09.017 30244078

[14] Shiraishi J, Li Q, Appelbaum D, Doi K. Computer-aided diagnosis and artificial intelligence in clinical imaging. Seminars in Nuclear Medicine. 2011; 41(6): 449–462. DOI: 10.1053/j.semnuclmed.2011.06.004 21978447

[15] Liang C-H, Liu Y-C, Wu M-T, Garcia-Castro F, Alberich-Bayarri A, Wu F-Z. Identifying pulmonary nodules or masses on chest radiography using deep learning: External validation and strategies to improve clinical practice. Clinical Radiology. 2020; 75(1): 38–45. DOI: 10.1016/j.crad.2019.08.005 31521323

[16] Maduskar P, Muyoyeta M, Ayles H, Hogeweg L, Peters-Bax L, van Ginneken B. Detection of tuberculosis using digital chest radiography: Automated reading vs. interpretation by clinical officers. The International Journal of Tuberculosis and Lung Disease. 2013; 17(12): 1613–1620. DOI: 10.5588/ijtld.13.0325 24200278

[17] Harris M, Qi A, Jeagal L, et al. A systematic review of the diagnostic accuracy of artificial intelligence-based computer programs to analyze chest x-rays for pulmonary tuberculosis. PloS One. 2019; 14(9). DOI: 10.1371/journal.pone.0221339 PMC6719854 31479448

[18] Park HH, Girdler-Brown BV, Churchyard GJ, White NW, Ehrlich RI. Incidence of tuberculosis and HIV and progression of silicosis and lung function impairment among former Basotho gold miners. American Journal of Industrial Medicine. 2009; 52(12): 901–908. DOI: 10.1002/ajim.20767 19882740

[19] Murray J, Davies T, Rees D. Occupational lung disease in the South African mining industry: research and policy implementation. Journal of Public Health Policy. 2011; 32(1): S65–S79. DOI: 10.1057/jphp.2011.25 21730995

[20] Stuckler D, Steele S, Lurie M, Basu S. Introduction: ‘Dying for gold’: The effects of mineral mining on HIV, tuberculosis, silicosis, and occupational diseases in Southern Africa. International Journal of Health Services. 2013; 43(4): 639–649. DOI: 10.2190/HS.43.4.c 24397231 PMC4524552

[21] Rees D, Murray J, Nelson G, Sonnenberg P. Oscillating migration and the epidemics of silicosis, tuberculosis, and HIV infection in South African gold miners. American Journal of Industrial Medicine. 2010; 53(4): 398–404. DOI: 10.1002/ajim.20716 19565628

[22] Smith J, Blom P. Those who don’t return: improving efforts to address tuberculosis among former miners in Southern Africa. New Solutions: A Journal of Environmental and Occupational Health Policy. 2019; 29(1): 76–104. DOI: 10.1177/1048291119832082 30791826

[23] Ehrlich R, Akugizibwe P, Siegfried N, Rees D. Silica exposure, silicosis and tuberculosis – a systematic review. BMC Public Health. 2021 in press. DOI: 10.1186/s12889-021-10711-1 PMC8136154 34016067

[24] Hnizdo E, Murray J. Risk of pulmonary tuberculosis relative to silicosis and exposure to silica dust in South African gold miners. Occupational and Environmental Medicine. 1998; 55(7): 496–502. DOI: 10.1136/oem.55.7.496 9816385 PMC1757613

[25] Solomon A. Silicosis and tuberculosis: Part 2—a radiographic presentation of nodular tuberculosis and silicosis. International Journal of Occupational and Environmental Health. 2001; 7(1): 54–57. DOI: 10.1179/oeh.2001.7.1.54 11210013

[26] Stuckler D, Basu S, McKee M, Lurie M. Mining and risk of tuberculosis in sub-Saharan Africa. American Journal of Public Health. 2011; 101(3): 524–530. DOI: 10.2105/AJPH.2009.175646 20516372 PMC3036676

[27] Ehrlich R. A century of miners’ compensation in South Africa. American Journal of Industrial Medicine. 2012; 55(6): 560–569. DOI: 10.1002/ajim.22030 22431163

[28] Maboso B, Moyo D, Muteba K, et al. Burden of disease among Basotho ex-miners in a large out-reach medical assessment programme. Occupational Health Southern Africa. 2020; 26(4): 145–152.

[29] Kistnasamy B, Yassi A, Yu J, et al. Tackling injustices of occupational lung disease acquired in South African mines: Recent developments and ongoing challenges. Globalization and Health. 2018; 14(60). DOI: 10.1186/s12992-018-0399-9 PMC6022447 29954399

[30] Tshiamiso Trust. Tshiamiso Trust. https://www.tshiamisotrust.com2021. Accessed March 5, 2021.

[31] Young C, Barker S, Ehrlich R, Kistnasamy B, Yassi A. Computer-aided detection for tuberculosis and silicosis in chest radiographs of gold miners of South Africa. International Journal of TB and Lung Diseases. 2020; 24: 444–451. DOI: 10.5588/ijtld.19.0624 32317070

[32] Laney A, Pontali E. Computer-assisted interpretation of chest radiographs: Signs of hope for silicosis and tuberculosis. The International Journal of Tuberculosis and Lung Disease. 2020; 24(4): 362–363. DOI: 10.5588/ijtld.19.0805 32317057 PMC7822060

[33] Yassi A, Spiegel J, Barker S. Improving efficiency of assessing (ex)miners for tuberculosis (TB) and silicosis: Innovations to promote social justice. http://med-fom-ghrp-spph.sites.olt.ubc.ca/files/2021/05/May-5th-2021-ayFinal.pdf. Accessed May 5, 2021.

[34] Liu V, Musen MA, Chou T. Data breaches of protected health information in the United States. JAMA: The Journal of the American Medical Association. 2015; 313(14): 1471–1473. DOI: 10.1001/jama.2015.2252 25871675 PMC4479128

[35] Canadian Association of Radiologists Artificial Intelligence Working Group. Canadian Association of Radiologists white paper on ethical and legal issues related to artificial intelligence in radiology. Canadian Association of Radiologists’ Journal. 2019; 70(2): 107–118. DOI: 10.1016/j.carj.2019.03.001 30962048

[36] Beauchamp T, Childress J. Principles of Biomedical Ethics, 7th Edition. New York, NY: Oxford University Press; 2013.

[37] Koplan JP, Bond TC, Merson MH, et al. Towards a common definition of global health. The Lancet. 2009; 373(9679): 1993–1995. DOI: 10.1016/S0140-6736(09)60332-9 PMC9905260 19493564

[38] Yassi A, Spiegel J, Barker S, Ehrlich R. Use of computer aided detection to support triage for efficiency at the MBOD. http://med-fom-ghrp-spph.sites.olt.ubc.ca/files/2021/05/Rodney-presentation-mining-may5.pdf. Accessed May 5, 2021.

[39] Khan FA, Pande T, Tessema B, et al. Computer-aided reading of tuberculosis chest radiography: Moving the research agenda forward to inform policy. European Respiratory Journal. 2017; 50(1700953). DOI: 10.1183/13993003.00953-2017 28705949

[40] Franzblau A, teWaterNaude J, Sen A, et al. Comparison of digital and film chest radiography for detection and medical surveillance of silicosis in a setting with a high burden of tuberculosis. American Journal of Industrial Medicine. 2018; 61(3): 229–238. DOI: 10.1002/ajim.22803 29210092

[41] Girdler-Brown BV, White NW, Ehrlich RI, Churchyard GJ. The burden of silicosis, pulmonary tuberculosis and COPD among former Basotho goldminers. American Journal of Industrial Medicine. 2008; 51(9): 640–647. DOI: 10.1002/ajim.20602 18566985

[42] Winfield AF, Jirotka M. Ethical governance is essential to building trust in robotics and artificial intelligence systems. Philosophical Transactions of the Royal Society A: Mathematical, Physical and Engineering Sciences. 2018; 376(2133). DOI: 10.1098/rsta.2018.0085 PMC6191667 30323000

[43] Republic of South Africa. Protection of Personal Information Act (POPI Act). https://popiacoza/2019-2021. Accessed May 5, 2021.

[44] Dwivedi YK, Hughes L, Ismagilova E, et al. Artificial Intelligence (AI): Multidisciplinary perspectives on emerging challenges, opportunities, and agenda for research, practice and policy. International Journal of Information Management. 2019; 101994. DOI: 10.1016/j.ijinfomgt.2019.08.002

[45] Topol EJ. High-performance medicine: The convergence of human and artificial intelligence. Nature Medicine. 2019; 25(1): 44. DOI: 10.1038/s41591-018-0300-7 30617339

[46] Coiera E. The fate of medicine in the time of AI. The Lancet. 2018; 392(10162): 2331–2332. DOI: 10.1016/S0140-6736(18)31925-1 30318263

[47] Canadian Institutes of Health Research (CIHR), Canadian Institute for Advanced Research (CIFAR). AI for Public Health Equity. 1 25, 2019.

[48] Sun TQ, Medaglia R. Mapping the challenges of Artificial Intelligence in the public sector: Evidence from public healthcare. Government Information Quarterly. 2019; 36(2): 368–383. DOI: 10.1016/j.giq.2018.09.008

[49] Lewis P. Sick miners to get up to R500k: Historic settlement reached in silicosis case. GroundUp. 5 3, 2018. https://www.groundup.org.za/article/historic-settlement-between-gold-industry-and-ex-mineworkers-lung-disease/.

[50] Mahajan A, Vaidya T, Gupta A, Rane S, Gupta S. Artificial intelligence in healthcare in developing nations: The beginning of a transformative journey. Cancer Research, Statistics, and Treatment. 2019; 2(2): 182. DOI: 10.4103/CRST.CRST_50_19

[51] Treviranus J. The three dimensions of inclusive design: A design framework for a digitally transformed and complexly connected society. University College Dublin; 2018.

[52] Racine E, Boehlen W, Sample M. Healthcare uses of artificial intelligence: Challenges and opportunities for growth Healthcare Management Forum 2019. Los Angeles, CA: SAGE Publications. DOI: 10.1177/0840470419843831 31234654

[53] Loh E. Medicine and the rise of the robots: A qualitative review of recent advances of artificial intelligence in health. BMJ Leader. 2018. DOI: 10.1136/leader-2018-000071

[54] Courtland R. Bias detectives: The researchers striving to make algorithms fair. Nature. 2018; 558(7710): 357–357. DOI: 10.1038/d41586-018-05469-3 29925973

[55] Campbell SH, Scott JT. Rousseau’s Politic Argument in the Discourse on the Sciences and Arts. American Journal of Political Science. 2005; 49(4): 818–828. DOI: 10.1111/j.1540-5907.2005.00157.x

[56] Horton R. Offline: The case against (and for) public health. The Lancet. 2016; 388(10060): 2578. DOI: 10.1016/S0140-6736(16)32387-X 27894652

